# Therapeutic Potential of Andrographolide Isolated from the Leaves of *Andrographis paniculata* Nees for Treating Lung Adenocarcinomas

**DOI:** 10.1155/2013/305898

**Published:** 2013-08-20

**Authors:** Yu-Tang Tung, Hsiao-Ling Chen, Hsin-Chung Tsai, Shang-Hsun Yang, Yi-Chun Chang, Chuan-Mu Chen

**Affiliations:** ^1^Department of Life Sciences, Agricultural Biotechnology Center, National Chung Hsing University, Taichung 402, Taiwan; ^2^Department of Bioresources, Da-Yeh University, Changhwa 515, Taiwan; ^3^Taichung Hospital, Department of Health, Taichung 403, Taiwan; ^4^Department of Physiology, National Cheng Kung University, Tainan 701, Taiwan; ^5^Department of Life Sciences, Office of Research and Development, National Chung Hsing University, No. 250, Kuo Kuang Road, Taichung 402, Taiwan

## Abstract

Andrographolide is one of the major diterpene lactones found in *Andrographis paniculata* Nees and exhibits remarkable inhibitory effects on various cancers. In this study, the antipulmonary cancer effects of andrographolide were studied in a lung tumor mouse model induced by human vascular endothelial growth factor *A*_165_ (*hVEGF*-A_165_). These results demonstrated that andrographolide significantly reduced the expression of hVEGF-A_165_ compared with a mock group in the Clara cells of the lungs. In addition, andrographolide also decreased tumor formation by reducing VEGF, EGFR, Cyclin A, and Cyclin B expression on the transcriptional and translational levels. These results indicated that andrographolide treatment on the overexpression of VEGF can arrest the cell cycle, which induced pulmonary tumors in transgenic mice. In conclusion, the antiangiogenesis and chemotherapeutic potential of andrographolide may provide a cure for pulmonary tumors in the future.

## 1. Introduction

According to the latest cancer mortality statistics from the People's Health Bureau of Taiwan, pulmonary cancer is ranked as a leading cause of Taiwanese cancer-related deaths [1]. Worldwide, lung cancer has been the most common form of cancer for several decades. Although chemotherapeutic agents for pulmonary tumors have developed, the lung cancer death rate is still high. Thus, many researchers have focused on early tumor detection and more active treatments as the tumor prevention methods [2]. For example, many researchers have discovered useful phytochemicals (compounds found in plants) and phytochemicals derivatives that provide novel anticancer therapies that have been successfully used in the clinic.

*Andrographis paniculata* Nees, a traditional medicine in Southeastern Asian countries, has been widely used in the clinic as an immunostimulant [3] and for the treatment of the common cold [4], myocardial ischemia [5], pharyngotonsillitis [6], and respiratory tract infections [7]. The major component of *A. paniculata* is andrographolide, which has been used to treat colds, diarrhea, fever, and inflammation, as well as infectious diseases [8]. In addition, the chemopreventive effects against and the inhibitory effects on cancer cell growth of andrographolide have been demonstrated in breast, colon, epidermoid, gastric, liver, leukemia, myeloma, peripheral blood lymphocytes, and prostate cancers [9–12]. The present study is the first to investigate whether andrographolide treatment could inhibit the formation of lung tumor in the hVEGF-A_165_ overexpressing transgenic mice. The airway hyperresponsiveness (AHR) was measured during a five-month treatment of mice with andrographolide. In addition, the pathological histology and the hVEGF immunohistochemistry staining of the lungs of these mice were examined. The related mRNA and protein expression in tumorigenesis and cell cycle regulator makers were also tested to evaluate the mechanism of tumor inhibition by treatment with andrographolide. 

## 2. Materials and Methods

### 2.1. Production of Lung Tumor Transgenic Mice

We have generated the mccsp-hVegf-A_165_-sv40 transgenic mice from pronuclear microinjection technique. Homozygous (hVEGF-A_165_^+/+^) or hemizygous (hVEGF-A_165_^+/−^) transgenic mice were identified by genomic DNA PCR. The genomic DNA isolated from the tails of the mice with the following primers: VEGF-94(+): 5′-AAGGAGGAGGGCAGAATCATC-3′ and VEGF-315(−): 5′-GAGGTTTGATCCGCATAATCTG-3′. The exogenic human VEGF-A_165_ protein and mRNA expression levels in homozygous (hVEGF-A_165_^+/+^) or heterozygous (hVEGF-A_165_^+/−^) transgenic mice were also detected by Western blot and RT-PCR.

### 2.2. Animals

**Figure 1:**
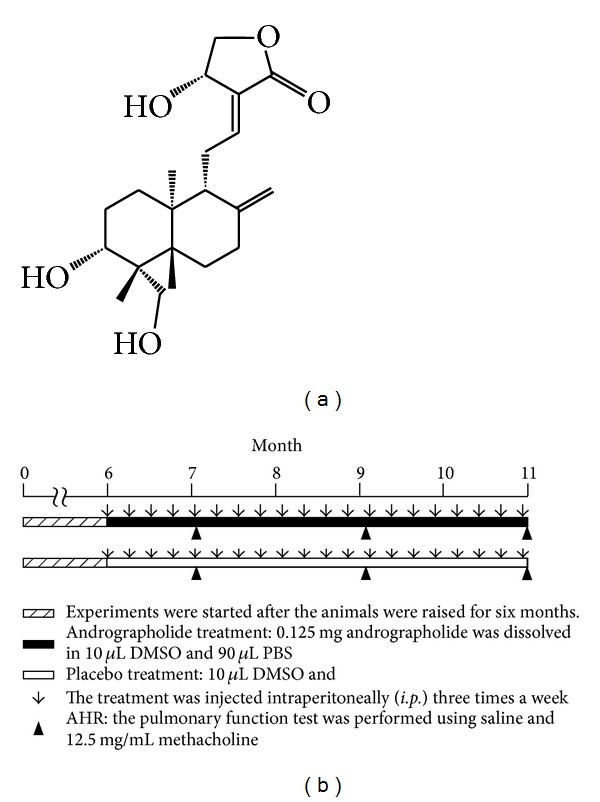
Active component of andrographolide (An) extracted from the leaves of *Andrographis paniculata* Nees and the animal trial timeline. (a) Chemical structure of andrographolide. (b) The schedule of andrographolide treatment. Mice were injected intraperitoneally (*i.p.*) with andrographolide (5 mg/kg body weight) three times a week for 5 months, and mice were sacrificed at 11 months.

A total of 12 male transgenic mice were given with free access to water and standard laboratory diet at a temperature- and humidity-regulated environment (22 ± 2°C, 65 ± 5% RH) with 12 h dark/light cycle. This study was conducted according to institutional guidelines and approved by the Institutional Animal Care and Utilization Committee of National Chung-Hsing, Taiwan (IACUC no. 96-83). The transgenic mice displaying the hemizygous (hVEGF-A_165_^+/−^) genotype were randomly divided into two groups (*n* = 6) based on their treatment: Tg/Mock (transgenic mice treated with a mock or placebo) and Tg/Andrographolide (transgenic mice treated with andrographolide). Andrographolide (An; Figure 1(a)) was obtained from Sigma Chemical (St. Louis, MO, USA) and dissolved in DMSO. Mice were injected intraperitoneally (*i.p.*) with andrographolide (5 mg/kg body weight) three times a week for 5 months, and mice were sacrificed at 11 months (Figure 1(b)). Pulmonary tissues were collected for pathological histology, immunohistochemistry staining, RNA extraction, and protein extraction according to our previously published protocols [13–15]. All the experiments were repeated twice.

### 2.3. Measurement of the Airway Hyperresponsiveness (AHR)

Bronchial provocation tests that evaluate the AHR in the Tg/Mock and Tg/Andrographolide groups were performed using methacholine. First, the basal pulmonary function was measured; then saline and methacholine at a concentration of 12.5 mg/mL were converted to aerosol using a nebulizer and allowed to be inhaled five times through a Rosenthal-French dosimeter. The pulmonary function was measured 30 times with a portable microspirometer for 3 minutes, and the enhanced pause (penh) values were selected to represent the pulmonary function. The penh, a dimensionless value, represents the proportion of maximal expiratory to maximal inspiratory box pressure signals and the timing of expiration. The AHR was presented as the penh in response to the increasing damage to pulmonary functionality [16].

### 2.4. Pathological Histology

Pulmonary tissues were fixed in 10% buffered formaldehyde (pH 7.0), embedded with paraffin, sectioned into 3 *μ*m sections, and examined using hematoxylin and eosin (H&E) staining. The hematoxylin and eosin (H&E) staining was described according to our previous reports [17, 18].

### 2.5. Immunohistochemistry Staining

IHC staining of pulmonary tissues, the VECTASTAIN ABC kit (Universal, Vector, USA) was used. Tissue sections (a thickness of 5 *μ*m) were placed on slides and incubated overnight at 4°C with rabbit anti-hVEGF-A monoclonal primary antibody as previously described [19]. Sections were developed using diaminobenzidine (DAB) as chromogenic substrates and counterstained with hematoxylin [20].

### 2.6. Real-Time RT-PCR

RNA was extracted from lung tissue using a Trizol reagent (Invitrogen, Carlsbad, CA) in accordance with the manufacturer's instructions. cDNA synthesis reaction was performed by PCR as described for ImProm-IITM reverse transcriptase (Promega, USA). Quantitative real-time RT-PCR (qRT-PCR) was carried out using SYBR Green in a Rotor-Gene 6000. The gene expressions of 14 genes (*vegf*, *kdr*, *nrp-1*, *myc*, *brca-1*, *mmp2*, *mmp9*, *egfr*, *erk2*, *survivin*, *cyclin a*, *cyclin b1*, *cyclin d,* and* cyclin e*) were performed on using cDNA from pulmonary tissue by qRT-PCR [21].

### 2.7. Western Blotting

The total protein of the pulmonary tissues from each mouse was homogenized in 500 *μ*L of RIPA buffer (5 mM Tris-HCl pH 7.4, 0.15 M NaCl, 1% NP40, 0.25% sodium deoxycholate, 5 mM EDTA, and 1 mM ethylene glycol-bis (2-aminoethyl-ether)-N,N,N,N-tetraacetic acid) and centrifuged at 12,000 ×g for 30 min at 4°C. Protein determination and Western blotting was performed according to the procedure reported [20, 22] with slight modifications. The protein samples (50 *μ*g) were resolved by a 10% SDS-PAGE and electrophoretically transferred to a PVDF membrane. Thus, the membrane was incubated with primary antibody (VEGF-A, EGFR, ERK2, Cyclin A, Cyclin B, or GAPDH) overnight at 4°C and further incubated for 1 h with anti-rabbit IgG antibody conjugated to HRP. The protein expressions were detected by the enhanced chemiluminescence reagents (ECL).

### 2.8. Statistical Analysis

Statistical significance of differences between treatments was determined by Student's *t*-test. *P* < 0.05 (*) or *P* < 0.01 (**) were considered to be statistically significant. 

## 3. Results

### 3.1. Effects of Andrographolide on the Airway Hyperresponsiveness (AHR) Parameters of Pulmonary Functionality

**Figure 2:**
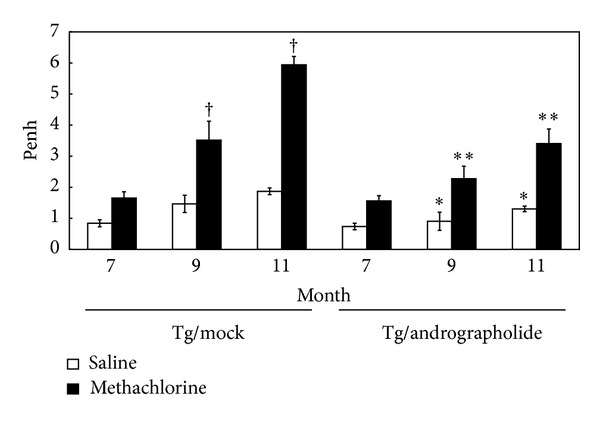
Effects of andrographolide on the airway hyperresponsiveness (AHR) parameters of pulmonary functionality. Data are presented as the means ± SEM (*n* = 6). ^†^ *P* < 0.01 Tg/Mock versus Tg/Mock group at 7 months. *P < 0.05 Tg/Andrographolide group versus Tg/Mock group in the same month. **P < 0.01 Tg/Andrographolide group versus Tg/Mock group in the same month.

The mccsp-hVegf-A165-sv40 transgenic mice which induces pulmonary tumor [16] was used as a lung cancer animal model for test of andrographolide (An) therapeutic effects. The animal trials timeline was showed in Figure 1(b). Several invasive and noninvasive methods have been used for the evaluation of the airway responsiveness of mice [23]. The AHR, a noninvasive procedure, enables easily and quickly obtained measurements, and it has been an important characteristic of pulmonary function. In this study, the AHR of the andrographolide or mock treatment was measured after 7, 9, and 11 months (Figure 2). The penh values of the Tg/Mock treatment with methacholine were significantly increased from 1.65 (7 months) to 3.52 (9 months), and 5.94 (11 months). However, 5 mg/kg body weight of andrographolide effectively reduced the penh elevation, exhibiting values of 1.57 (7 months), 2.28 (9 months), and 3.41 (11 months). This result revealed that andrographolide dramatically reduced the damage to pulmonary functionality.

### 3.2. Effect of Andrographolide Treatment on Lung H&E Staining and Pathological Analysis

**Figure 3:**
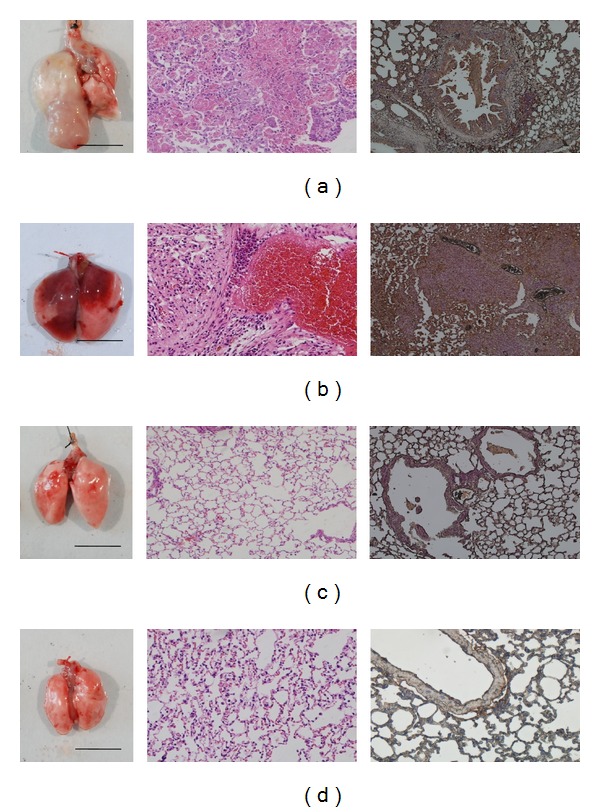
The exterior, histopathological slides and immunohistochemical (IHC) staining of the lung tissues of 11-month-old transgenic mice that overexpress hVEGF-A_165_ in the (a, b) Tg/Mock group and (c, d) Tg/Andrographolide group following 5 months of andrographolide treatment.

Figure 3 and Table 1 indicate that transgenic mice (83.3%; 5/6) have found pulmonary tumors; these tumors primarily consisted of neoplasms growth on the periphery of the pulmonary alveolus and adenomas growing on the site near the lung bronchus. In the pulmonary alveoli of the lung bronchi of the transgenic mice, some obvious large-grained pink cells, which represent macrophages, are indicative of an inflammatory response. Furthermore, the group treated with 5 mg/kg andrographolide decreased the neoplasm growing on the periphery of the pulmonary alveolus and the adenomas growing near the site of the lung bronchus (Figures 3(c) and 3(d)). This pathological analysis illustrated that andrographolide has antipulmonary cancer potential (33.3%; 2/6) compared with Tg alone (83.3%; 5/6) and induces anti-inflammatory responses in the lungs of transgenic mice that overexpress hVEGF-A_165_.

### 3.3. Effect of Andrographolide Treatment on Immunohistochemistry Staining

Angiogenesis is required for the tumor formation, tumor growth, invasion, and metastasis. VEGF is a principal regulator of vasculogenesis and angiogenesis. Using immunohistochemistry staining (IHC staining), we observed that VEGF was overexpressed in the Clara cells of lung tissues in the transgenic mice (Figures 3(a) and 3(b)) and that the treatment with andrographolide significantly reduced the expression levels of VEGF (Figures 3(c) and 3(d)). These results indicate that transgenic mice treatment with andrographolide can decrease VEGF expression compared with those that were mock treated; thus it may reduce the new blood vessel formation and growth. The anti-VEGF therapy of andrographolide may prevent the previously observed tumor growth, invasion, and metastasis. 

### 3.4. Andrographolide Suppresses the Marker Genes for Tumor Formation

**Figure 4:**
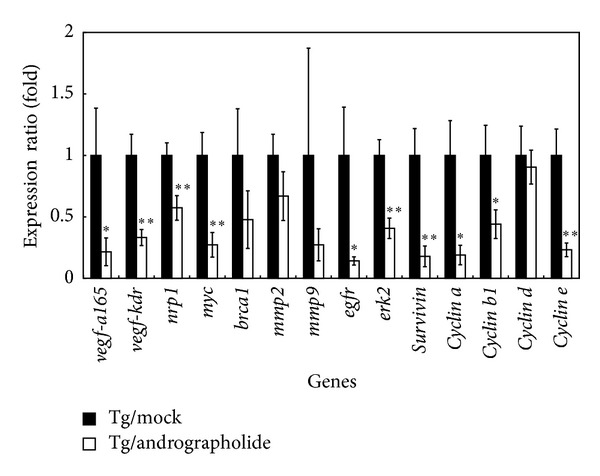
Real-time PCR validations of mRNA expression levels of *vegf*, *kdr*, *nrp-1*, *myc*, *brca-1*, *mmp2*, *mmp9*, *egfr*, *erk2*, *survivin*, *cyclin a*, *cyclin b1*, *cyclin d,* and* cyclin e* in the lung tissues of the Tg/Mock and Tg/Andrographolide groups. *β*-Actin was used as an internal control. The quantitative mRNA expression levels were measured by qRT-PCR. Data are presented as the means ± SEM (*n* = 6). *P < 0.05 versus Tg/Mock group. **P < 0.01 versus Tg/Mock group.

The mRNA levels of *vegf-a165*, *kdr*, *nrp-1*, *myc*, *brca-1*, *mmp2*, *mmp9*, *egfr*, *erk2*, *survivin*, *cyclin a*, *cyclin b1*, *cyclin d,* and* cyclin e* in the Tg/Mock and Tg/Andrographolide groups were evaluated using qRT-PCR (Figure 4). These results demonstrated that treating Tg mice with 5 mg/kg of andrographolide significantly decreased the expression of *vegf-a165*, *kdr*, *nrp-1*, *c-myc*, *egfr*, *erk2*, *surviving*, *cyclin a*,* cyclin b1,* and *cyclin e*. *Kdr* and its coreceptor *nrp-1*, which are the main angiogenic receptors in the vegf pathway, are downregulated. In addition, both the c-Myc and Ras/MEK/ERK pathways play a vital role in the progression of the G1-cell cycle phase by enhancing cyclins. Thus, in this study *erk2 and c-myc* down-regulation may induce cell cycle G1 arrest. Furthermore, *cyclin a* is essential for *c-myc*-modulated cell-cycle progression. 

### 3.5. Andrographolide Suppresses Cell Cycle Progression Signaling Pathways

**Figure 5:**
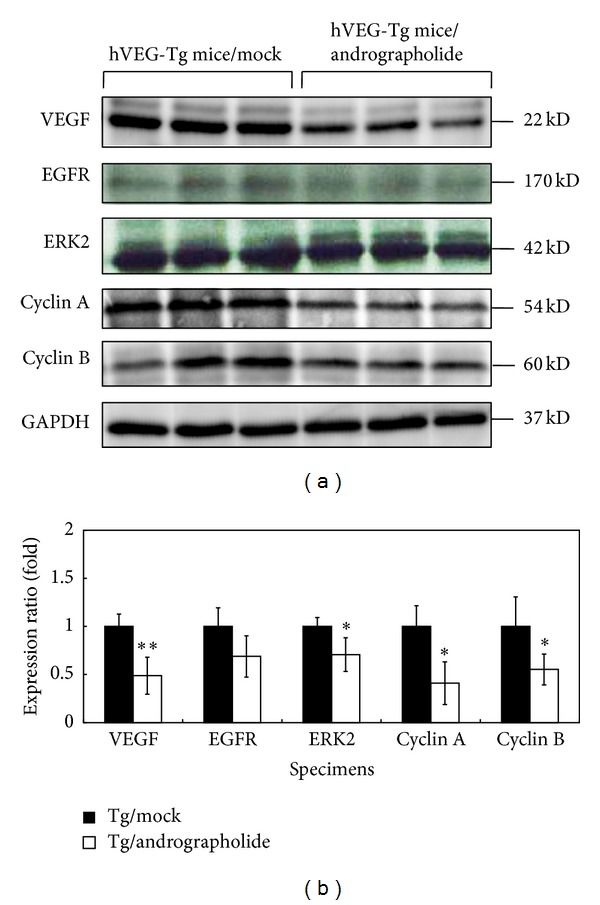
Western blot analysis of VEGF-A, EGFR, ERK2, Cyclin A, and Cyclin B protein in the lung tissues of the Tg and Tg/Andrographolide groups. GADPH was used as an internal control. Data are presented as the means ± SEM (*n* = 6). *P < 0.05 versus Tg group. **P < 0.01 versus Tg group.

VEGF, ERK2, Cyclin A, and Cyclin B were significantly decreased in the Tg/Andrographolide group compared with the Tg/Mock group using Western blot analysis (Figure 5). Andrographolide affects the cell cycle by reducing the levels of Cyclin A and Cyclin B, which regulate the S and G2/M phases, respectively. The andrographolide-induced downregulation of Cyclins A and B may decrease the progression of cells through the S and G2/M phases. This result indicates that the protein expression involved in the S to M phase transition in transgenic mice was inhibited by andrographolide.

## 4. Discussion

Previous studies have shown that *Andrographis paniculata* possesses potent antiatherosclerotic, anti-inflammatory, antioxidant, hepatoprotective, immunomodulatory, and anticancer effects [24–29]. Andrographolide is one of the major diterpene lactones found in *A. paniculata* and displays potent anticancer and immunomodulatory effects, which may lead to its development as a chemotherapeutic agent [28, 30]. In addition, many researchers have demonstrated that andrographolide displays excellent anticancer effects on breast, colon, epidermoid, gastric, HeLa, liver, leukemia, myeloma, peripheral blood lymphocytes, and prostate cancers [9–12]. Many *in vitro* studies demonstrated that andrographolide promotes apoptosis in cancer cells, suppresses cancer cell proliferation, and induces cell-cycle arrest [12, 31].

The stabilization of HIF-1*α* mediated the overexpression of VEGF, which has been identified as a common step in the development of carcinomas. Thus, VEGF-inhibitors, which directly or indirectly target the tumor vasculature, have become common in anticancer therapies [32]. In the present study, we use 11-month-old transgenic mice that overexpress a lung-specific hVEGF-A_165_. These mice contain the mccsp-hVegf-A_165_-sv40 poly(A) transgene for researching pulmonary tumors and serve as an animal model for researching the regulatory mechanism of andrographolide. We found that these transgenic mice are susceptible to the growth of pulmonary tumors. However, following a 5-month treatment with andrographolide (5 mg/kg b.w. three times a week), these mice exhibited a dramatic decrease in the formation of solid tumors when compared with the mock treatment. The histological examination also indicated that the andrographolide treatment reduced pulmonary tumor formation and inflammation (Figure 3). 

Initially, the cancer cells continue to proliferate and thus create a nutrition and oxygen deficiency, which causes significant cell death. The secretion of large quantities of VEGF-A_165_ induces vasculogenesis to provide the rapidly growing tumor with sufficient quantities of nutrients and oxygen [33]. This solid tumor growth is dependent on angiogenesis, and thus the suppression of tumor blood vessels offers a new alternative to prevent and treat cancer. Using IHC staining, we observed that andrographolide reduced the expression of hVEGF-A_165_ to normal levels, specifically in the Clara cells in the lungs of transgenic mice. These results indicate that andrographolide reduced the expression of VEGF in Clara cells when compared with the mock treatment. VEGF is essential for vasculogenesis and angiogenesis during development and tumor progression. In addition, Zhao et al. [34] demonstrated that andrographolide effectively inhibited VEGF expression in prostate cancer. Lin et al. [1] found that the inhibition of PI3 K/Akt signaling by andrographolide significantly suppressed the protein and mRNA levels of HIF-1*α*, as well as the protein level and transcriptional activation of VEGF in A549 cells. In this study, we further found that treatment with andrographolide in lung tumor animal model effectively reduced the expression of VEGF in tumor tissue; this activity demonstrates that andrographolide may prove a potent antiangiogenic agent for the treatment of pulmonary cancer.

**Figure 6:**
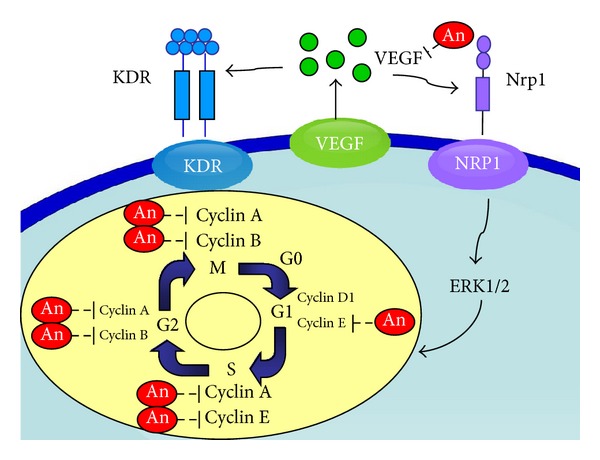
Scheme of the andrographolide regulatory pathway. The effects of andrographolide inhibition of lung tumors are hypothesized through a cell-cycle signaling pathway. The diagram shows that andrographolide may inhibit VEGF-A, KDR, Nrp1, ERK2, Cyclin A, and Cyclin B expression.

In addition, andrographolide can reduce pulmonary functional damage (Figure 2) and prevent pulmonary cancer formation through elimination of VEGF, ERK2, Cyclin A, and Cyclin B proteins (Figure 5). Andrographolide induced the reduction of Cyclin A and Cyclin B, which likely resulted in the reduced progression through the S and G2/M phases. These results indicated that andrographolide suppressed the protein expression at the transition from the S-phase to the M-phase in transgenic mice. These results also demonstrate that the inhibition of the cell cycle may be a vital pathway that andrographolide affects the transgenic mice that overexpress VEGF. Furthermore, Satyanarayana et al. [12] revealed that andrographolide inhibits cell-cycle arrest in MCF-7 cells by inducing the expression of p27 (the cell-cycle inhibitory protein) and decreasing the expression of cyclin-dependent kinase. Shi et al. [31] also suggested that andrographolide can induce cell-cycle arrest at the G1/S phase by CKI-cyclin-Cdk signaling in Lovo cells. These results revealed that andrographolide can induce cell-cycle arrest in cancer cells. Furthermore, we found that *erk2 *downregulation was followed by a downstream decreasing of *c-myc *expression that may induce cell cycle arrest in the G1 phase. Additionally, *cyclin a* is essential for *c-myc*-modulated cell-cycle progression in pulmonary cancer formation. Finally, Tsai et al. [18] found that andrographolide inhibited chemotic migration, which may be due to the inhibition of the ERK1/2 and Akt protein kinase cascades. Here we show a relationship between andrographolide treatment and the transcriptional and translational levels of VEGF, EGFR, ERK2, and Cyclin A (Figure 6).

## 5. Conclusion

The andrographolide-induced inhibition of VEGF by arresting the cell cycle may be a critical mechanism for preventing pulmonary tumor angiogenesis and metastasis. The inhibition of VEGF by andrographolide may be a useful method for the chemoprevention of pulmonary cancer. 

## Figures and Tables

**Table 1:** Lung tumorigenesis frequency of Tg/Mock and Tg/Andrographolide groups in the mouse lung tissues (*n* = 6) using histopathological image analysis.

Variable	Tg/Mock	Tg/Andrographolide
Normal	0 (0%)	2 (33.3%)
Cyst	6 (100%)	4 (66.7%)
Damaged alveoli	4 (66.7%)	2 (33.3%)
Mild emphysematous change	0 (0%)	3 (50%)
Prominent emphysematous change	6 (100%)	1 (16.7%)
Hemosiderin-laden macrophages in alveoli	2 (33.3%)	0 (0%)
Old hemorrhage	6 (100%)	2 (33.3%)
Moderate lymphocytic infiltration	0 (0%)	1 (16.7%)
Marked chronic lymphoid infiltration	6 (100%)	2 (33.3%)
Neoplasm	3 (50%)	1 (16.7%)
Lymphoma	6 (100%)	1 (16.7%)
Adenocarcinoma	5 (83.3%)	2 (33.3%)

## Abbreviations

AHR: Airway hyperresponsiveness
An: Andrographolide
DAB: Diaminobenzidine
H&E: Hematoxylin and eosin
IHC: Immunohistochemistry staining
Penh: The enhanced pause
qRT-PCR: Quantitative real-time RT-PCR
VEGF: Vascular endothelial growth factor.
